# Use of the *K*-Nearest Neighbour Classifier in Wear Condition Classification of a Positive Displacement Pump

**DOI:** 10.3390/s21186247

**Published:** 2021-09-17

**Authors:** Jarosław Konieczny, Jerzy Stojek

**Affiliations:** Department of Process Control, Faculty of Mechanical Engineering and Robotics, AGH University of Science and Technology, 30-059 Krakow, Poland; koniejar@agh.edu.pl

**Keywords:** learning system, classifier, *K*-nearest neighbours, diagnostics, signal analysis, multi-piston pump, vibrations

## Abstract

This paper presents a learning system with a *K*-nearest neighbour classifier to classify the wear condition of a multi-piston positive displacement pump. The first part reviews current built diagnostic methods and describes typical failures of multi-piston positive displacement pumps and their causes. Next is a description of a diagnostic experiment conducted to acquire a matrix of vibration signals from selected locations in the pump body. The measured signals were subjected to time-frequency analysis. The signal features calculated in the time and frequency domain were grouped in a table according to the wear condition of the pump. The next step was to create classification models of a pump wear condition and assess their accuracy. The selected model, which best met the set criteria for accuracy assessment, was verified with new measurement data. The article ends with a summary.

## 1. Introduction

Diagnosis of failures in elements and systems of power hydraulics is based on analysing signals received from transducers located in typical places of these elements. Commonly used ones include vibration, pressure and flow rate signals, and thermal signals.

Based on a review of ongoing research work involving diagnostics of hydraulic systems, there are three main [1] trends in the construction of such systems that can be identified.

As the first, we can distinguish systems based on the model of the diagnosed object, e.g., by using an extended Kalman filter [2] as a state observer. Such a solution was used in certain papers [2,3] for the detection of failures of axial displacement pumps, which consisted of the evaluation of leaks developing in piston–cylinder pairs of these pumps. On the other hand, the paper by [4] used an adaptive Kalman filter algorithm to detect piston leakage in a hydraulic cylinder. Another example includes using a sliding observer with adaptive amplification to reconstruct errors (failures) of an electrohydraulic positioning system described in the paper [5].

The second diagnostic system is based on signal analysis and extracting relevant features from the signals to identify a fault in the monitored component or hydraulic system. Time-frequency analysis [6], wavelet analysis, or the Hilbert–Huang transform are used here.

The paper by [7] describes the application of the continuous wavelet transform to detect a modelled hydraulic cylinder malfunction, consisting of degradation of the cylinder piston rod seals and accompanying external leakage. Additionally, the paper explores the possibility of isolating this malfunction from another type of actuator failure—internal leakage. The issue of detecting internal leakage in a hydraulic cylinder was also addressed in [8]. The use of the Hilbert–Huang transform (HHT) is described here as an effective tool for detecting such faults. On the other hand, one paper [9] discusses the application of improved adaptive multiscale morphology analysis (IAMMA) to demodulate damage signals of piston shoes of a multi-piston pump. 

The third type of system encountered for diagnosing hydraulic systems is the so-called intelligent fault identification system. Many studies on intelligent fault identification have been carried out and successfully applied to hydraulic system diagnostics [10,11,12]. The machine learning method used in hydraulic brake condition monitoring is described in [13]. A support vector machine (SVM) was used here as a fault classification algorithm. On the other hand, one paper [14] presents the possibility of detecting abrasive damage of piston shoes of an axial piston pump by using a new classifier, the so-called extreme learning machine (ELM). In addition, the paper describes and compares three methods for extracting diagnostic features from measured signals. 

The use of deep machine learning in device monitoring problems with a discussion of architecture variants based on a Boltzmann machine, deep belief network (DBN), convolutional neural networks (CNN), and recurrent neural networks (RNN) is presented in [15].

A deep machine learning method using deep belief networks (DBNs) architecture for fault classification in axial piston pumps is presented in [16]. An experimental study was conducted to detect and classify the four most common axial piston pump faults. The classification accuracy rate was 97.40%, confirming the feasibility and effectiveness of detecting multiple faults in axial piston pumps using deep belief networks (DBNs).

Multi-piston pumps are essential in high-power hydraulic systems. The proper functioning of the hydraulic system as a whole often depends on its correct operation. Wear and tear of individual pump components usually leads to a decrease in pump operating pressure and an increase in volumetric losses, which subsequently leads to a decrease in overall pump efficiency, an increase in vibration, and an increase in pump noise.

Much of the work on diagnosing malfunctions in positive displacement pumps is based on pre-prepared failures of pump components, which are then incorporated into their design. This approach does not provide a complete picture of damage development and is only an approximation. The article’s authors used a different approach based on obtaining the wear of elements of the tested pump naturally based on many hours of its operation under the assumed load at a lower oil cleanliness class. In addition to obtaining the wear of the pump components naturally, the presented method allows the development of damage and the accompanying symptoms to be controlled.

The foregoing review of papers on the diagnostics of hydrostatic systems shows that most authors carrying out performance assessment for such systems used signals measured in stationary conditions of operation (after the system had reached thermal stabilisation). However, it is known from operational practice that the information contained in the signals measured under conditions of non-stationary pump operation (with changing viscosity of the working fluid) provides a complete picture of the state of wear of its elements. The authors of this article used the signals measured in the entire operating range of the pump, i.e., in stationary and non-stationary conditions of its operation, to assess the wear state of the tested pump.

In this paper, the authors present a machine learning system [17] to classify the wear state of a multi-piston positive displacement pump. In engineering applications, machine learning systems have many advantages, including the ability to build a system with good classification accuracy using a reasonable amount of learning data and short learning times for the developed diagnostic models. From the beginning, it was assumed that the learning system would be based on vibration signals measured at distinct locations in the pump body and additional signals from static and dynamic pressure transducers mounted in the discharge port obtained from a passive diagnostic experiment.

According to the authors, the original approach of this work is:

- to demonstrate that the use of a basic classification algorithm such as KNN is sufficient to obtain a classifier with high accuracy for recognising the wear state of a positive displacement pump;
- preparation and execution of a research experiment in such a way as to obtain the wear of pump elements naturally based on many hours of operation at a lower oil cleanliness class;
- to use signals measured in the entire range of operation of the pump (i.e., in stationary and non-stationary conditions of its operation) to assess the state of wear of the tested pump.

## 2. Description of the Research Object

**Figure 1:**
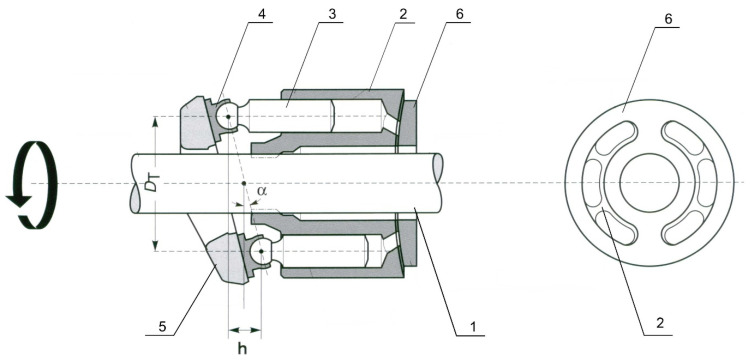
Simplified design diagram of a swashplate axial piston pump: 1—shaft, 2—rotor, 3—piston, 4—sliding shoe, 5—swashplate, 6—valve plate [20].

The research object consisted of an axial multi-piston pump [18,19] with a swash plate; the simplified constructional scheme presented in Figure 1. In this type of pump design, the rotor (2) and the piston assembly (3) are mounted coaxially on the drive shaft (1). The pistons perform a rotary motion together with the rotor. In addition, the shoes (4) of the pistons cooperating with the swashplate (5) deflect at a certain angle *α* with respect to the axis of the pump rotor.

Wear and tear of positive displacement pump components is caused both by forces that occur during the cooperation of the individual parts forming kinematic pairs (e.g., piston–cylinder, valve plate–rotor, piston shoe–swashplate), as well as by unsuitable operating conditions of the pump, including exceeding the nominal working pressure of the pump, operation with too-low viscosity of the working medium, and lack of or insufficient filtration of the working medium. 

The most common type of wear on positive displacement pump components is abrasive wear [21,22,23]. Excessive load on the rotor assembly leads, among other things, to abrasive wear of its parts and increased radial clearance in the piston–cylinder pairs. This results in increased volumetric losses and reduced overall pump efficiency.

Wear of the swashplate, which interacts with the shoe surfaces of the rotor pistons, leads to the formation of an elliptical depression on its surface and working it entirely out. This reduces the mechanical–hydraulic efficiency of the pump. In turn, the working out of the valve plate is caused, among other things, by the loss of the lubricating layer between the disc surface and the rotor face surface. This wear results in the appearance of flow microchannels on the surface of the disc bridge. The resulting channels cause the working fluid to flow between the suction and discharge zones of the pump, causing a lack of tightness and a reduction in the operating pressure and volumetric efficiency of the pump.

## 3. Course of the Study

**Figure 2:**
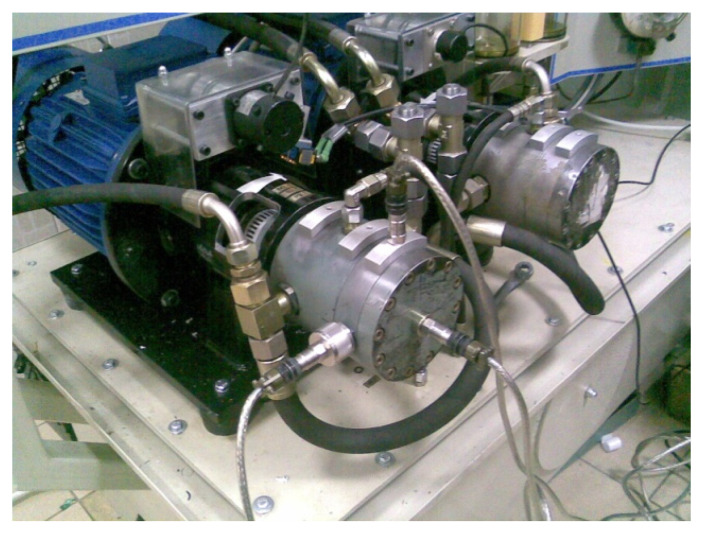
View of the tested pump on the laboratory station.

Studies on the wear development of the multi-piston pump components were conducted on a purpose-built laboratory station. One of the main objectives of the study was to obtain the wear of the pump components naturally. Therefore, the tests carried out were multi-hour tests with actual operating conditions of the pump. During the study experiment, diagnostic signals from the assembled measuring transducers were continuously recorded: the flow rate of the working medium, static and dynamic pressure, and vibration acceleration of the pump body. Measurements of the vibrating body acceleration were carried out for three measurement axes (X, Y, Z) after the transducers were previously mounted on the pump body near the valve plate, rotor, and swashplate [6]. Signals of 1 s in length were recorded at intervals of 15 minutes between measurements. The daily duration of the experiment was 10 h of pump operation under static load. The sampling rate of the signals was 50 kHz. A view of the pump test system with the vibration transducers installed is shown in Figure 2.

After laboratory testing, the pump was evaluated for the wear of its components. Wear was found on the rotor and valve plate assembly based on visual evidence. No wear was found on the surface of the swashplate or the surface of the piston shoes. The lack of wear on this kinematic pair was due to the static operating conditions of the pump under which the lubricating film between the mating surfaces of the swashplate and the piston shoe did not disappear.

**Figure 3:**
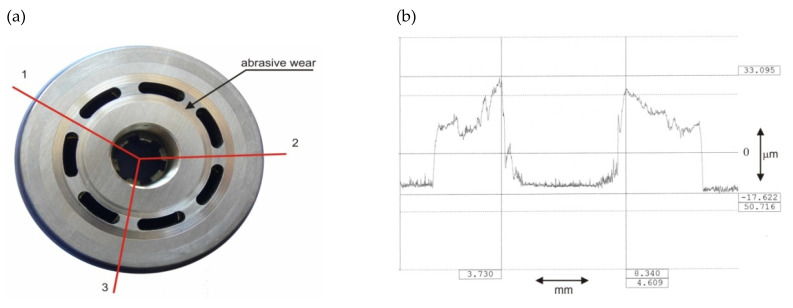
View of worn rotor face and its wear profile: (**a**) worn rotor face: 1,2,3 wear measurement directions, (**b**) wear profile.

The observed wear of the rotor assembly consisted of an average increase of about 10 μm in radial clearance in each piston–cylinder pair. In addition, wear was found on the rotor face mating with the valve plate surface. Rotor face degradation was measured using a contact profilometer [24] in three selected measurement directions (Figure 3). Virtually uniform rotor face wear was found when comparing the measured profile runs, with an average wear depth of about 50 µm.

**Figure 4:**
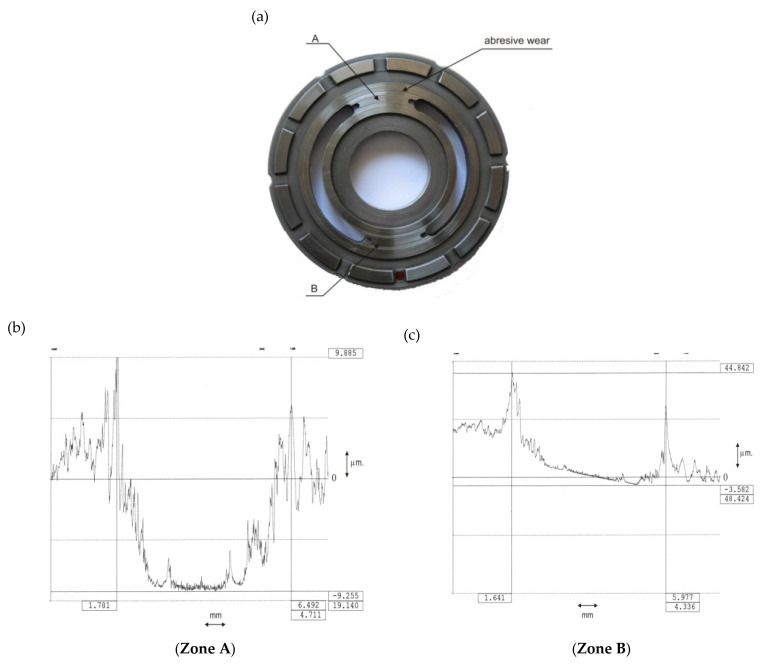
View of the worn valve plate surface and the wear profiles of the transition zones: (**a**) valve plate, (**b**) zone A wear profile, (**c**) zone B wear profile.

The wear of the valve plate surface resulted in the development of flow microchannels on the surfaces of the transition zones (the so-called bridges) between the suction and delivery ducts (Figure 4). Non-uniform wear of the transition zones was found after measuring the profiles on transition zones A (transition from suction to delivery side) and B (transition from delivery to suction side) (Figure 4). The surface of transition zone A (transition from the suction side to delivery side) degraded more. 

The pump body vibrations obtained from the experiment were grouped according to the state of its efficiency, with the following classes (labels) assigned: *efficient* *pump*, *end of service*, and *worn out pump*. 

The operating condition of the pump was monitored by changing its output pressure. By comparing the pressure values day by day for the individual measurements, it was assumed that if the pressure drop at the pump outlet reached 10%, the pump remained efficient (label: “efficient pump”). Further increases in pump outlet pressure drop (up to 20% compared to the beginning of the recording) were classified as a pump near the end of service status (label: “end of service”). A pump outlet pressure drop of more than 20% was treated as a worn pump (label: “worn pump”). 

The next stage of the study was to create models of pump efficiency classification based on previously grouped vibration waveforms.

## 4. Learning System

Modelling multi-piston pumps is one of the most difficult in mechanics due to the complexity of physical phenomena occurring during their operation (usually strongly nonlinear and non-stationary). Existing mathematical models are usually approximations of the phenomena occurring in pumps during their operation [25]. Learning systems, which model the state of an industrial process or its component (e.g., a machine) based solely on available measurement data assigned to the process state (class), are becoming increasingly common in maintenance engineering. 

For learning techniques, learning systems can be divided into learning systems that use supervised learning and learning systems in which unsupervised learning occurs [26,27].

Supervised learning algorithms will be used in the wear condition classification problem of a multi-piston positive displacement pump.

### 4.1. Data Preparation for the Learning System

The recorded measurement signals obtained after each day of the experiment were subjected to qualitative evaluation to remove possible significant errors resulting, among others, from unexpected faults (interferences) while recording. Then, the mean value was removed from the signals and subjected to high-pass filtering with a cut-off frequency of f = 20 kHz. Taking advantage of the fact that while measuring the signals, the pump shaft rotation marker signal was also measured, each of the recorded signals was divided according to it. Because the signals (recorded every 15 min) were 1 s long, the 25 splits of each measured signal with a duration of one complete revolution (i.e., 0.04 s) were obtained. In this way, signal matrices of one rotation length were obtained. During the experiment, along with the recording of diagnostic signals, the signals of the temperature rise of the pump body and the temperature of the working fluid in the tank were recorded. An example of the change in pump body temperature (and working fluid temperature) from one day of testing is shown below.

**Figure 5:**
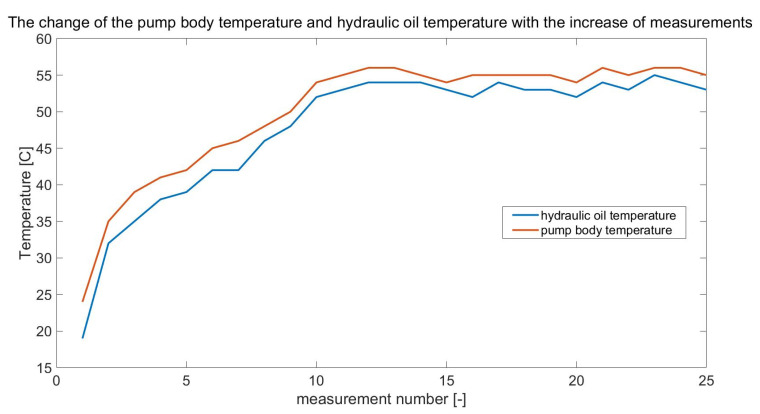
The change in the pump body temperature and hydraulic oil temperature with the increase in measurements.

From the presented runs (Figure 5), it can be seen that the recording of physical quantities in the first part of the research experiment takes place under non-stationary conditions. Once the thermal stabilisation of the liquid has been achieved at T = 50 °C, the rest of the investigation proceeds at a constant temperature at which the viscosity of the oil does not change and can be taken as constant. It was assumed that the input data for the learning system would be the data obtained from the measurements of physical quantities over the entire operating range of the pump, i.e., in stationary and non-stationary conditions of its operation. 

A total of 429 runs of pump body vibration signals were recorded, of which 288 (144 each) were signals measured for a pump in service (label: “operational pump”) and a pump in transition condition (label: “end of service”). The last 141 runs were obtained from the worn pump operation (label: “worn-out pump”). The next stage of data preparation for the learning system was its division into data used to learn the classifier model and data intended for its later validation. It was assumed that 25% of the overall data would be used to test the validity of the obtained classifier model. The data prepared in this way were loaded into the workspace of the Matlab package [28,29], where further analysis was carried out, consisting of:

- selecting and calculating appropriate signal features;
- selecting the classifier model;
- evaluating the effectiveness of the classifier used.

### 4.2. Selection of Signal Features

Another important consideration in developing a learning system was the selection of signal features on which the system would be based. 

Time-domain and frequency domain signal features were determined for each of the obtained pump body vibration signal matrices [30]. In determining the features of signals in the time domain, it was decided to determine their variability and the amount of information contained in them. The standard deviation and entropy [31] were used as measures of these features. Additionally, the RMS values were determined. The entropy of the signal spectrum, the maximum power spectral density PSD value, and the frequency at which the maximum power spectral density occurred were taken as the characteristics of signals in the frequency domain. A total of 56 features was obtained.

The calculated features of the vibration signals were grouped in a table, the last column of which were the classes (labels) of the pump’s operating condition, i.e., “*operational pump*”, “*end of service*”, and “*worn-out pump*”.

Theoretically, the number of calculated signal features (which are the input data for the system) is unlimited. Yet, practically, the aim is to obtain a minimum number of features that describe the properties of the tested object well. This promotes a compact model with a good fit.

Optimisation of selecting appropriate features to evaluate the wear rate of a multi-piston pump was performed using the minimum redundancy maximum relevance (MRMR) algorithm [32]. This algorithm determines the optimal—given a pump state classification *y*—set *S* of features *x* and *z*, maximising their applicability and minimising redundancy *W*_S_. The algorithm is based on the pairwise computed mutual information:

- *I*(*x, z*) of features and the mutual information;
- *I*(*x, y*) of features and pump condition.

(1) $$V_{s}=\frac{1}{\left|S\right|}\underset{x\in S}{\sum }I\left(x,y\right)$$

(2) $$W_{s}=\frac{1}{{\left|S\right|}^{2}}\underset{x,z\in S}{\sum }I\left(x,z\right)$$

where:

- *V*_s_—the applicability of features from set *S*;
- *W*_s_—the redundancy of features from set *S*;
- |S|—The number of features in set *S*;
- *I*(*x*, *y*)—the calculated value of the mutual information of feature *x* and condition *y* of the pump;
- *I*(*x*, *z*)—the calculated value of mutual information of features *x*, *z* from set *S.*

The mutual information value in Equation (3) of features *x* and *z* in set *S* was calculated based on their probabilistic joint distribution *p*(*x,y*) and the respective marginal probabilities *p*(*x*) and *p*(*y*) using the adaptive algorithm described in [33]. The mutual information value *I*(*x*, *y*) of the *x* features and the condition *y* of the pump was calculated in the same way.

(3) $$I\left(x,y\right)=\underset{i,j}{\sum }p\left(x_{i},y_{j}\right)\log{\frac{p\left(x_{i},y_{j}\right)}{p\left(x_{i}\right)p\left(y_{i}\right)}}_{}$$

where:

- *p*(*x,y*)—the joint probabilistic distribution of *x* and *y*;
- *p*(*x*)—the marginal probability of *x*;
- *p*(*y*)—the marginal probability of *y*.

Finding the optimal set *S* of features (among the full set Ω of all 56 listed features) that minimises their redundancy and simultaneously maximises their applicability requires an algorithm that computes the value of the mutual information coefficient *MIQ_X_* (which is the quotient of a feature’s applicability to its redundancy). Feature classification is based on selecting those with the largest *MIQ_X_* coefficient value (in practice, larger than the assumed cut-off value).

(4) $$\begin{array}{c} max\\ x\in S^{c} \end{array}MIQ_{X}=\begin{array}{c} max\\ x\in S^{c} \end{array}\frac{I\left(x,y\right)}{\frac{1}{\left|S\right|}\sum_{z\in S}I\left(x,z\right)}$$

**Figure 6:**
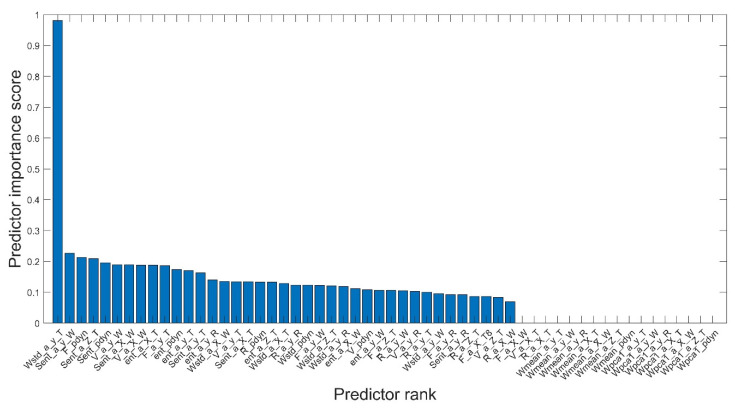
Ranking of features that meet the condition of most significant applicability with minimum redundancy—based on data obtained under stationary and non-stationary pump conditions.

The significance classification of the features obtained from the measurements of the physical quantities of the pump is shown graphically in Figure 6.

Out of 56 features (obtained from measurements in stationary and non-stationary conditions of the pump’s operation), the ones with the value of the calculated *MIQ*_X_ coefficient greater than 0.2 were selected to assess the pump’s wear condition. The features [31] that satisfied the foregoing condition are included in Table 1.

After extracting the most significant signal features for assessing pump wear condition, the next step was to check pump wear condition prediction accuracy by the simplified model (using the determined features) compared to the full model (using all 56 calculated features). For this purpose, an appropriate classification algorithm (classification model) was selected, and a null hypothesis was formulated and evaluated using a statistical multi-pass test. 

### 4.3. Selection of Classification Algorithm

In systems using supervised learning and those with unsupervised learning, there is a large group of learning algorithms, the selection of the most appropriate of which depends on numerous factors. First of all, to choose a proper learning algorithm, the task that the model is supposed to perform (classification, regression, clustering) must be well defined. Another issue is the type and size of the input data, which affects the learning rate, the memory load on the computer (controller), and the prediction accuracy of the output (model response). Choosing the correct algorithm is not straightforward, and only an experienced operator (long-term researcher) can quickly identify the suitable algorithm. Usually, selecting the best classification algorithm is done based on multiple trials of each type and evaluating the obtained classifiers in terms of speed of operation, classification accuracy, and memory load of the counting unit.

In the group of algorithms that satisfy the issue of wear classification of a multi-piston pump, one can distinguish [17]:

- Decision Trees

In this algorithm, a decision tree structure is used to classify data based on a starting point (start) and branches that are a binary decision system, the final branches of which are the result of assigning data to a class.

- Discriminant Analysis

This is based on the analysis of Gaussian distributions of signals from a set of observations (inputs). The classifier estimates the parameters of the Gaussian distribution from the observations, and based on these, assigns them to the appropriate class.

- Support Vector Machines

These classify data by finding the best hyperplane that separates the data of one class from another. The best hyperplane is assumed to be the one that separates the data by the largest margin.

- *K*-Nearest Neighbours Classifiers

These determine the affiliation of new data from the input set to a specified class based on the position of an assumed number (*K* number) of the input set’s nearest (neighbouring) data to these data. Therefore, measuring the distance of the classified data from its neighbouring data is taken as the measure of location.

- Naive Bayes Classifiers

These represent a probabilistic classifier in which the input variables are (naively) assumed to be mutually independent. Using Bayes’ theorem, this classifier calculates the probabilities of input data belonging to a specific class. 

The verification of the accuracy of the pump wear assessment by the simplified model in relation to the full model (using all 56 features of the measured signals) was based on the use of the *K*-Nearest Neighbours classifier [34,35]. The *K*-Nearest Neighbours (KNN) algorithm is a simple, easy-to-implement supervised machine learning algorithm that can be used to solve both classification and regression problems. This type of classifier has minimum model learning time, average classification time, and average classification accuracy. Moreover, the KNN classifier is usually used when the size of the data set to be classified is small or average, as in the case of engineering issues (such as the classification of the operating condition of a positive displacement pump).

The following *H*_o_ null hypothesis was accepted:

A full model using all 56 features of the measured signals classifies the wear condition of a multi-piston pump as accurately as a simplified model using the most significant extracted features.

A multivariate (5 × 2) student’s *t* test with a random distribution of signals was used to verify the null hypothesis. As a measure of the accuracy of the pump wear condition classification by the full model and the simplified model, the value of the fitting error coefficient *e* defined by the equation was used:

(5) $$e=\frac{\sum_{j=1}^{n_{test}}w_{j}I\left(\hat{p_{1j}}\neq y_{j}\right)}{\sum_{j=1}^{n_{test}}w_{j}}$$

where:

- *n_test_*—the number of observations;
- *w_j_*—the weight of the *j* observation;
- *I*(*x*)—the function tag, taking the value 1 for the truth of the assumption, otherwise taking 0;
- $\hat{p_{1j}}$—the recognised pump condition for the first model at the *j* observation;
- $y_{j}$—the actual state of the pump at the *j* observation.

The results of evaluating the accuracy of the developed models based on the features determined from the measured signals are presented below.

When comparing the pump wear condition classification results by the full and simplified models, the obtained evaluation of the hypothesis was zero (h = 0 with probability value *p* = 0.57). This indicated that the null hypothesis cannot be rejected; thus, a simplified model could be used to classify the wear condition of the pump. The estimated classification errors of the full model (*e*_1_ error) and the simplified model (*e*_2_ error) from the multiple repeat test are summarised in the tables below (Table 2 and Table 3).

### 4.4. Classification of the Pump Wear Condition

**Figure 7:**
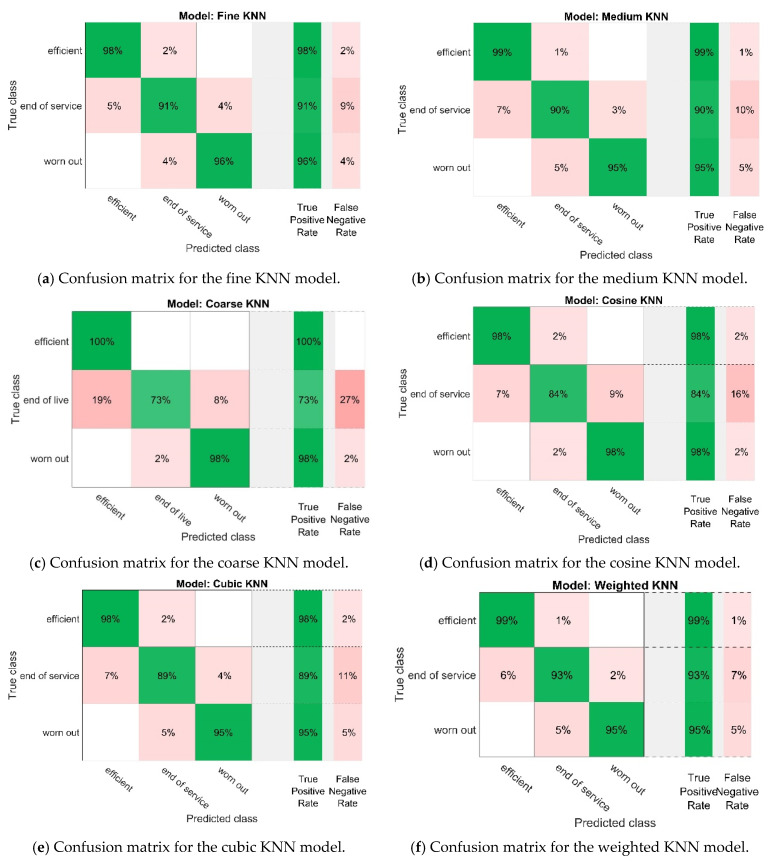
Confusion matrices of the wear condition classification obtained from models using the *K*-Nearest Neighbours classifier.

The classification of the pump wear condition was performed by simplified models using the *K*-Nearest Neighbours classifier [36]. The models differed in the number of *K*-Nearest Neighbours adopted and the type of distance measure used. The accuracy of the obtained models was evaluated by means of cross-validation. For the analysis, the input data were divided into five equal intervals, successively used as test sets and the remaining ones as learning sets. The mean error was then calculated as an assessment of the accuracy of the models obtained. The correctness of the recognition of the pump state by the models was shown by the confusion matrices, which are summarised in Figure 7.

The accuracy of pump wear condition classification by the determined models, along with their main features, is summarised in Table 4.

Analysing the features of the models summarised in Table 4, it was found that almost all the models had good accuracy in classifying the wear state of the pump, and the accuracy of two of them—fine KNN and weighted KNN—was the highest (94.5%). The misclassification cost of these models was also the lowest and amounted to 47%. The main difference between these models was the number of selected neighbours in the KNN algorithm: one in the fine KNN model and 10 in the weighted KNN model. 

Aiming at the physical realisation of the diagnostic system based on the machine code generated from the selected classification model (e.g., C, C++) requires selecting a model that is distinguished by the highest accuracy of pump state prediction and the lowest classification error rate at the same time. Comparing the model accuracies summarised in Table 4, it was found that this condition is met by the first model (fine KNN) and the last model (weighted KNN). The main difference between these models was the number of selected neighbours. Bearing in mind that in the case of using a KNN algorithm in a real diagnostic system, the selection of a larger number of neighbours usually results in higher classification accuracy (especially when the analysed signal is noisy), the choice of the weighted KNN model is a better solution. In addition, comparing the classification matrices of these models (Figure 7) showed that the weighted KNN model recognises individual pump operating conditions with slightly higher accuracy than the fine KNN model. Therefore, the weighted KNN model was selected for further analysis.

Verification of the effectiveness of the adopted weighted KNN model was carried out on the signals recorded during the experiment, which were not previously used for its determination. A matrix of 90 signals was prepared (30 signals for each pump operating state). Then the features of these signals were calculated, and the weighted KNN model was verified. The classifier model based on pre-calculated features of the measured signals recognised pump efficiency classes with 90% accuracy.

**Figure 8:**
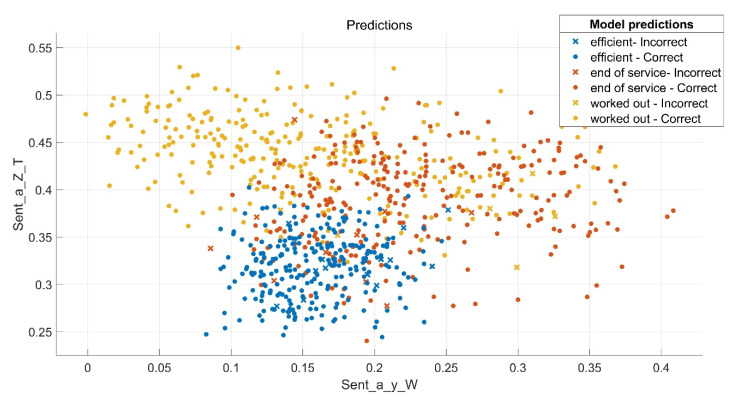
Example of prediction for entropy distribution of pump swashplate vibration signals as a function of entropy of pump rotor signals using the weighted *K*-Nearest Neighbours model.

An example of entropy distribution prediction of pump swashplate vibration signals (on measuring directions Y and Z) obtained using a verified model (weighted KNN) is shown in Figure 8.

## 5. Summary

In terms of classifying damages of machines and devices, the operation of which is characterised by occurrence of complex mechanical and fluid phenomena (as it is in the case of the analysed positive displacement pump), careful preparation of input data for the selected classifier of their wear condition is an important factor. The right choice of signals and where to measure them creates the potential for highly informative data. In the case of positive displacement pump classification, the input data sets containing the measured signals should be as large as possible. The input data must include runs from operation over the entire range of operating pressure variation, and the viscosity of the working medium changing with temperature. This affects the better training of the obtained classifier model and the obtained efficiency in classifying the wear condition of the pump. Another important consideration is the selection of signal features that best separate machine performance or failure classes. The minimum number of features used in the classification process affects the learning time of the classifier and prevents overtraining.

In classifying the degree of efficiency of a multi-piston pump, the features that best separated it were the standard deviation of the vibration acceleration signal measured at the pump swashplate and the entropies of the power spectral density of the pump body vibration acceleration signals. The applied basic *K*-Nearest Neighbours algorithms confirmed their suitability for the classification of the wear condition of a multi-piston positive displacement pump with good accuracy (above 90%) of the correct diagnosis of its operating condition. Improving the classification accuracy of the wear condition of the tested pump will require retraining the obtained classifier models using a larger number of recorded measurement signals.

## Figures and Tables

**Table 1:** The most significant signal features for assessing pump wear condition.

Property	*MIQ_X_* Coefficient Value
*Wstd_a_y_T*	0.98
*Sent_a_y_W*	0.23
*F_pdym*	0.22
*Sent_a_z_T*	0.21
*Sent_pdyn*	0.2

Where *Wstd_a_y_T*—standard deviation of the vibration acceleration signal measured at the pump swashplate in the Y direction, *Sent_a_y_W*—power spectral density entropy of the vibration acceleration signal measured at the pump rotor in the Y direction, *Sent_a_z_T*—power spectral density entropy of the vibration acceleration signal measured at the pump swashplate in the Z direction, *Sent_pdyn*—power spectral density entropy of the dynamic pressure signal, and *F_pdym*—frequency of the maximum power spectral density (PSD) of the dynamic pressure signal.

**Table 2:** Calculated *e*_1_ error values of pump wear condition classification for the full model using 56 signal features.

5 × 2 *t* Test	1	2
1	0.027	0.009
2	0.016	0.016
3	0.021	0.023
4	0.018	0.013
5	0.018	0.039

**Table 3:** Calculated *e*_2_ error values of pump wear condition classification for a simplified model using the 5 most relevant signal features.

5 × 2 *t* Test	1	2
1	0.025	0.023
2	0.025	0.027
3	0.032	0.018
4	0.023	0.032
5	0.034	0.03

**Table 4:** Pump state classification models and their main features.

Model	Fine KNN	Medium KNN	Coarse KNN	Cosine KNN	Cubic KNN	Weighted KNN
Number of variables	1	10	100	10	10	10
Misclassification cost	47	52	88	62	56	47
Distance metric	Euclidean	Euclidean	Euclidean	Cosine	Minkowski	Euclidean
Distance weight	Equal	Equal	Equal	Equal	Equal	Squared inverse
Accuracy [%]	94.5	94	89.8	92.8	93.5	94.5

## Data Availability

Data sharing is not applicable.

## Nomenclature

*V*_S_	applicability of features from set *S*
*W*_s_	redundancy of features from set *S*
|S|	number of features in set *S*
*I*(*x*, *y*)	value of the mutual information of feature *x* and condition *y* of the pump
*I*(*x*, *z*)	value of mutual information of features *x, z* from set *S*
Ω	set of all features
*y*	state classification
*x*, *z*	features
*MIQ_X_*	mutual information coefficient
*p*(*x,y*)	joint probabilistic distribution of features *x* and *y*
*p*(*x*)	marginal probabilities of *x*
*p*(*y*)	marginal probabilities of *y*
*e*	fitting error coefficient
*n_test_*	number of observations
*w_j_*	weight of the *j* observation
*I*(*x*)	function tag, takes the value 1 for the truth of the assumption, otherwise takes 0
$\hat{p_{1j}}$	recognised pump condition for the first model at the *j* observation
$y_{j}$	actual state of the pump at the *j* observation
*S^c^*	*c* features with non-zero relevance and zero redundancy in set *S*
